# Secondary Use of Laboratory Data: Potentialities and Limitations

**DOI:** 10.30699/ijp.2019.95692.1942

**Published:** 2019-08-01

**Authors:** Massoud Hajia

**Affiliations:** Research Center of Reference Laboratories, Health Reference Laboratory of Iran, Ministry of Health and Medical Education, Tehran, Iran

**Keywords:** Laboratory data, Scientific report, Data integration

## Abstract

Clinical databases have been developed in recent years especially during the course of all medical concerns including laboratory results. The information produced by the diagnostic laboratories have great impact on health care system with various secondary uses. These uses are sometimes as publishing new extracted information of laboratory reports which have been widely applied in the scientific journals. Nowadays, some large scale or national databases are also formed from the integration of these data from smaller centers in the field of human health in many countries. These databases are beneficial for different stakeholders who may need this information.

Unfortunately, reviewing some of these uses has indicated lots of errors in quality control, test validity, uniformity and so on. More importantly, some of the diagnostic procedures have been applied in the clinical diagnostic laboratories without even preliminary clinical evaluation studies. Therefore, any taken conclusion from these analyzed data may not be reliable.

This use requires checking the several specifications that have been notified in this study. Current review also intends to show how the correct information should be to extract for the scientific reports, or integrated in large scale databases.

## Introduction

Large clinical databases are becoming increasingly available every day to researchers as more hospitals and practices adopt electronic record systems (1). These records may cover a range of clinical fields, including infectious and non-infectious diseases with all specific and non-specific tests (2, 3). 

Analyzed information can be used in the process of patient care (i.e. diagnosis of diseases, treatment, screening and prevention). Analysis of laboratories data consequently yields information that is necessary for the evidence-based planning and decision making in the health care system that can be in a higher levels of hospital or university, even countrywide. 

Another issue is the accessibility that should be easy for every stakeholder, while new technologies are now more and more implemented in the clinical settings. Besides, accessibility of the data can be through the entire health sector. To accomplish this point, some critical points should be taken into consideration, such as reliability and accuracy of the diagnostic tests, correctly preparation of the patients’ reports, and standard formatting of the released information. Furthermore, having effective management system is important to manage the flow of information between health care providers, patients, and laboratories. These data are becoming increasingly important for the various stakeholders because of having clinical information that are collected over the years.

**Quality of Reports**

Before answering this question that how far laboratory databases can be used and expanded, it is necessary to provide required conditions for achieving reliable answers from research questions. The existence of poor quality data in the clinical information systems presents a challenge for the patients’ health. It will also affect the secondary uses of electronic health record data. Reliability and the accuracy of the test must be measured before any diagnostic test to be used for the patient’s diagnosis. The sensitivity and specificity of each laboratory test can be measured, which depend on the incidence rate of the infectious agent. Furthermore, the quality assurance program must be applied in the whole process of the diagnostic laboratories from receiving specimens up to reporting and even later steps. Therefore, it is necessary to have regular auditing in the clinical laboratories to achieve the standard accreditation (9). The quality of specimens needs to be precisely controlled to ensure of laboratory results. The reliability of the laboratory results are also dependent on the validity of the selected methods and the quality of equipment (i.e. devices, kits, reagents and other consumables) as well as on the quality assurance program (10). All technical activities must be implemented to obtain correct results according to the standard operational procedures (SOPs). It should be also noticed that the personnel have an important role in the quality of laboratory results (11). Finally, all kind of laboratory activities which are effective on the quality of test results should be continually monitored so as to any nonconformities be detected. This is important that corrective actions must be correlated to accomplish with subsequent preventive measures. Participation in the proficiency testing is the last necessary step that must be followed at least each semiannually (12, 13). As a result, these laboratory data will be valuable when are reported from accredited clinic center or clinical laboratory. 

These centers, whose results are used as part of a clinical trial, must be previously validated for their required accreditation. The results of research tests should not be integrated in the main clinical database (14, 15). Unfortunately, majority of the scientific journals are not attended to this point in those submitted papers which used such data. Unfortunately, we are observing that limited numbers of referees focus on the reliability methods of collected data, or on the use of accreditation of procedures.

**Laboratory Information Systems**

The development of information systems in the clinical laboratory field has greatly affected all aspects of the lab activity. This system plays an important role in the clinical laboratories operation for the utilization of and archiving laboratory tests results. 

An effective management system is necessary to manage the laboratory information intelligently for all the relevant stakeholders to access required data. The main mission of such program is managing the workflow and delivering the data to the centers who have requested. The laboratory information system provides a mechanism for electronically integration of the patient data to be used for the facilitation laboratory workflow processes. It is also suitable for the diagnostic interpretations aid in the quality assurance, and can be connected with other information systems. Data entering into the system is a critical step at this level. Any error at this level will directly affect the quality of the results (15).

Most laboratory errors are reported in the pre-analytical process specifically in the collection and handling of specimens, although some of them are observed in the post-analytical step (10). Therefore, it is essential to have an analytical quality control system that monitors the precision and accuracy of the deter-minations. It can be based on the periodic analysis of the sample control, or comparing the results of some diagnostic laboratories. 

Many analyzers have also the quality control prog-rams included in the management software. On the basis of different algorithms, the program warns to the user for any possible error. Once the results are prepared in available database, before becoming visible to the outside of the laboratory, it must be validated by a legally-qualified laboratory supervisor (16). Under-standing the reasons that cause false-positive and false-negative results is quite necessary toward more accurate decisions on the test material effects. Reports can be released whenever approved. This validation serves as the last filter for detecting possible errors (17). Errors can be related to the test units or reference intervals that lead to misinterpretation of the laboratory results as it is previously discussed. However, other errors are related to transcription. Transcription errors are among the most common laboratory errors. These errors happen when a correct result is produced from the checked procedure, but a mistake happens in the preparation of the patients’ report (18). 

The laboratory reports should be prepared in a standard format and contain all the essential information that is necessary for the proper understanding and interpretation. Reporting format might differ depending on the target group (i.e. report preparing for patients, physicians, other care providers or public health authorities) which may contain different relevant information (13).

**Secondary Use of Laboratory Results**

It has been observed in some scientific reports that researchers used laboratory data without any standardization. Therefore, the reported results lack the validity and reliability. We are going to explain how this information can be used to provide valuable results. Regarding selected topics, researchers may have several questions in mind. Preliminary aim of the diagnostic laboratory databases is to provide the standard conditions for the main duty of the laboratory that is reporting patients` results. No one expects that these databases be able to provide perfect answers for all the questions by the researchers. Those researchers who have enough experiences on the selected topics may have further queries. Hence, they are able to define a hypothesis for their questions. Research questions must be exactly defined. The answers may not be easily obtained from this available information. Therefore, specific parts of the collected data need to be selected for the proper answer. The selected data must have same specifications. All final results must be obtained in similar laboratory conditions (including methods, materials, equipment and so on). Then selected topic requires to be preliminarily evaluated for its potentially clear answers. It may need to change the group studied, or increase its number, even though it may be required the selected hypothesis to be amended. The step can be repeated a few times (19-22). 

Selected data needs to be transferred to the separate spreadsheet for later analysis, although some main issues need to be considered. All the unwanted and unclear cases need to be eliminated in the new file. Clinical data are heterogeneous across and within the information systems. The content must be homogenous and uniform to reach the purpose of research. Typical data quality issues encountered include: inaccurate data, incomplete data required for the operations or decisions, and inconsistencies across data sources. Once the pre-liminary processing was completed, all the names that represented a single concept must be similar and standard vocabularies (23). 

Researchers, who have followed these steps and reached a new finding, need to ensure of other aspects of the standard reporting. We are occasionally facing other problems in a few scientific reports. When the reports lead to a new finding, the authors emphasize just on the presentation of their results. It is very important that all the details of the quality control methods and the sources of the used materials be carefully mentioned. From this point of view, looking at some reports will not be useful (24-27). In these studies, the method of receiving patients` specimens and their quality has not been mentioned. Now, if we look carefully at the published reports, we find out that in many of these articles there are ambiguities about the method of testing and the quality of the materials tested and so on.

In Recent years, another type of secondary use of clinical records has been broadly applied. This new usage is integration of the local information to the large scale database for a wider survey. Therefore, all the selected data requires reconfiguration according to the new database. This data may have other stakeholders in a region or even a country, for instance, the prediction of the epidemic diseases, and supplying required pre-scription or other laboratory materials.

## Discussion

Several This survey clearly shows that routine clinical reports are valuable if all processes are accredited accurately. It is also important that the whole collected data to be checked at the first step by a supervisor who is professional specialist. These analyzed results should be definitely confirmed and interpreted by those who have enough experiences in this regard. They should be competent in the analytical and clinical aspects of the test. Interpretation helps to ensure that the significance of the clinical result is apparent and their analytic limitations have been revealed. 

The reports are helpful for most experts, if they explain the outcome of the patients` reports. These explanations are obviously based on the analytical and clinical performance characteristics of the tests and also are in correlation with the clinical settings in which the tests have been applied. It would be regardless of whether the report is a stand-alone or part of a large scale laboratory test done on the same specimens. Therefore, interpretations or any other recommendations need to be included in the reports by a laboratory professional (28-30). Composing such reports requires technical knowledge as well as medical expertise; sometimes it is indicating how other diagnostic processes or clinical parameters should be investigated. Furthermore, clinical interpretations offer new understanding of the laboratory findings. 

Hence with these considerations and supervision on any database, other stakeholders can access to it. This accessible data will obviously help the researchers and other medical specialists to upgrade their knowledge and provide better patient`s care and treatment. All the other stakeholders in the larger areas can achieve required information for the prediction of any epidemic disease or better treatment if we could design extra deeper program to cover all information of similar centers for further analysis. Today, these integrated clinical databases are being introduced as digital surveillance (31). A good example of such use is a successful reported project in Denmark. A nationwide database has been prepared for enabling real-time surveillance of the communicable diseases. It has been applied for the microorganisms as well as providing nationwide access for the healthcare personnel to microbiology reports. The aim of this project is to provide a close collaboration between all stakeholders including suppliers of laboratory information systems, clinical users and Danish political decision makers (32).

## Conclusion

Any error associated with the laboratory testing or data entry causes the collected information to be untrustworthy. Therefore, the necessary quality assurance program of the whole process must be performed under supervision of the expert technical knowledge. Furthermore, these centers must be regularly audited since continuous evaluation is crucial in the addressing system problems and creating user awareness of the system potentials. Then, providing scientific reports can be possible with the standard supervision on the laboratory record or clinical data. 

This opportunity can also be provided to make the information more widely available to other stakeholders. Also, it is essential that the basic required measures in the diagnostic labs be performed, if we want to benefit from this information to provide a broader database.

## References

[1] Marshall J, Chahin A, Rush B Review of Clinical Databases. MIT Critical Data, Secondary Analysis of Electronic Health Records" First Online: September 2016, Chapter 2.

[2] Debra Revere D, Hills RH, Dixon BE, Gibson PJ, Graniss SJ (2017). Notifiable condition reporting practices: implications for public health agency participation in a health information exchange. BMC Public Health.

[3] Collen MF, Slack WV, Bleich HL, Collen M, Ball M (2017). Medical Databases and Patient Record Systems. The History of Medical Informatics in USA.

[4] Hajia M, Rahbar M, Farzami MR, Asl HM, Dolatyar A, Imani M (2015). Assessing clonal correlation of epidemic Vibrio cholerae isolates during 2011 in 16 provinces of Iran. Curr Microbiol.

[5] Mafi M, Hajia M, Goya MM (2016). A five years study on the epidemiological approaches of Cholera in Iran. Caspian J Inter Medicine.

[6] Hajia M, Rahbar M, Fallah F, Safadel N (2012). Detection of Bordetella pertussis in infants suspected to have whooping cough. The open respiratory medicine journal..

[7] Khoo SH, Hajia M, Storey CC, Klapper PE, Wilkins EG, Denning DW, Dunbar EM, Corbitt G, Mandal BK (1998 May). Influenza-like episodes in HIV-positive patients: the role of viral and'atypical'infections. AIDS (London, England).

[8] Hajia M, Rahbar M, Amini R (2009). Is PCR assay reliable for diagnosis of extrapulmonary tuberculosis?. African Journal of Microbiology Research.

[9] Dahim P, Amini R, Safadel N (2009). Implementation of Quality Management System in Iranian Medical Laboratories. Iran J Public Health.

[10] Aisu S, Nyegenye W, Kiyaga C, Dfendu M, Sewanyana I, Namakula A, (et al) (2016). Quality assurance as an integral component of diagnostic testing in clinical laboratories and point-of care testing: the Uganda experience: country profile. African Journal of Laboratory Medicine.

[11] Ravaghi H, Khodayari Zarnaq R, Adel A, Badpa M, Adel M, Abolhassani N (2014). A Survey on Clinical Governance Awareness Among Clinical Staff: A Cross-Sectional Study. Global Journal of Health Science.

[12] Anjarani S, Safadel N, Dahim P, Amini R, Mahdavi S, Samiee S (2013). Establishment of national laboratory standards in public and private hospital laboratories. Iranian J Public Health..

[13] Safadel N, Anjarani S, Farzami MR, Amini R, Samiee SM, Dahim P (2014). Establishing an Iranian medical laboratory standard based on ISO 15189. Accreditation and Quality Assurance.

[14] Hajia M, Safadel N, Samiee SM, Dahim P, Anjarani S, Nafisi N (2013). Quality Assurance Program for Molecular Medicine Laboratories. Iran J Public Health.

[15] Grannis SJ (2011). Electronic Laboratory Data Quality and the Value of a Health Information exchange to support public health reporting processes. AMIA Annu Symp Proc.

[16] Hajia M, Sohrabi M Comment on “Upgrading the Iranian national laboratory standard to ISO 15189:2012” Submitted.

[17] Safadel N, Dahim P, Anjarani S, Rahnamaye Farzam Mi, Mirab Samiee S, Amini R (2013). Challenges of implementing Iranian national laboratory standards. Iranian J Public Health.

[18] Hajia M, Sohrabi A Current Situation of Molecular Diagnostic Assay in Iran: is it necessary to revise of health surveillance system at laboratory level? Submitted.

[19] Abhyankar S, Demner-Fushman D, McDonald CJ (2012). Standardizing clinical laboratory data for secondary use. Journal of Biomedical Informatics.

[20] Dixon BE, Siegel JA, Oemig T, Grannis S (2013). Electronic Health Information Quality Challenges and Interventions to Improve Public Health Surveillance Data and Practice. Electronic Health Information Quality.

[21] Bean NH, Martin SM (2001 ). Implementing a network for electronic surveillance reporting from public health reference laboratories: an international perspective. Emerg Infect Dis.

[22] Jonnalagadda SR, Goyal P, Huffman MD (2015). Automating data extraction in systematic reviews: a systematic review. Systematic Reviews.

[23] Dixon BE, Grannis SJ, Revere D (2013). Measuring the impact of a health information exchange intervention on provider-based notifiable disease reporting using mixed methods: a study protocol. BMC Medical Informatics and Decision Making.

[24] Hajia M, Amirzargar A, Ghoreish M, Sam M (2012). Estimation the frequency of human immunodeficiency virus among male and female patients, Iran. Cell Journal.

[25] Hajia M, M RahbarM, Alikhani Y (2005). Efficiency of PCR Method for Screening Pulmonary Tuberculosis Patients under DOTs Protocol Therapy. Iranian Journal of Public Health.

[26] Shafiei R, Mahmoodzadeh A, Hajia M, Sanati A, Shafiei F (2011). Epidemiology of malaria in Khorasan Razavi province, northeast of Iran, within 7 years (April 2001-March 2008). Ann Trop Med Publ Health.

[27] Rahbar M, Zahraei M, Omidvarnia A, Afshani MT, Glami M, Sabourian R (2010). Survey of epidemiology and bacteriology features of cholera in Iran. Asian Pac J Trop Med.

[28] Wadhwa V, Rai S, Thukral T (2012). Laboratory quality management system: Road to accreditation and beyond. Indian J Med Microbiol.

[29] Gulley ML (2007). Clinical Laboratory Reports in Molecular Pathology. Arch. Of Path & Lab Medicine.

[30] Silk BJ, Berkelman RL (2005). A Review of Strategies for Enhancing the Completeness of Notifiable Disease Reporting. Journal of Public Health Management & Practice.

[31] Morse SS, Mazet JA, Woolhouse M, Parrish CR, Carroll D, Karesh WB, Zambrana-Torrelio C, Lipkin WI, Daszak P (2012). Prediction and prevention of the next pandemic zoonosis. The Lancet.

[32] Voldstedlund M, Haarh M, Mølbak K (2014). The Danish microbiology database (MiBa) 2010 to 2013. Eurosurveillance.

